# Investigating the Role of Antigen Orientation on the Immune Response Elicited by *Neisseria meningitidis* Factor H Binding Protein on GMMA

**DOI:** 10.3390/vaccines10081182

**Published:** 2022-07-26

**Authors:** Renzo Alfini, Brunella Brunelli, Erika Bartolini, Martina Carducci, Enrico Luzzi, Francesca Ferlicca, Scilla Buccato, Barbara Galli, Paola Lo Surdo, Maria Scarselli, Giacomo Romagnoli, Elena Cartocci, Domenico Maione, Silvana Savino, Francesca Necchi, Isabel Delany, Francesca Micoli

**Affiliations:** 1 GSK Vaccines Institute for Global Health (GVGH), 53100 Siena, Italy; renzo.x.alfini@gsk.com (R.A.); martina.x.carducci@gsk.com (M.C.); francesca.x.necchi@gsk.com (F.N.); 2 GSK, 53100 Siena, Italy; brunella.x.brunelli@gsk.com (B.B.); erika.x.bartolini@gsk.com (E.B.); enrico.x.luzzi@gsk.com (E.L.); francesca.x.ferlicca@gsk.com (F.F.); scilla.x.buccato@gsk.com (S.B.); barbara.x.galli@gsk.com (B.G.); losurdopaola7@gmail.com (P.L.S.); maria.x.scarselli@gsk.com (M.S.); giacomo.x.romagnoli@gsk.com (G.R.); elena.x.cartocci@gsk.com (E.C.); domenico.x.maione@gsk.com (D.M.); silvana.x.savino@gsk.com (S.S.); isabel.x.delany@gsk.com (I.D.)

**Keywords:** GMMA, outer membrane vesicles, *Neisseria meningitidis*, conjugation

## Abstract

GMMA are outer membrane vesicles (OMVs) released from Gram-negative bacteria genetically modified to enhance OMVs formation that have been shown to be optimal systems to enhance immunogenicity of protein antigens. Here, we selected *Neisseria meningitidis* factor H binding protein (fHbp) and used the conjugation chemistry as a tool to alter antigen orientation on GMMA. Indeed, fHbp was randomly linked to GMMA or selectively attached via the N-terminus to mimic native presentation of the protein on the bacterial surface. Interestingly, protein and peptide array analyses confirmed that antibodies induced by the selective and the random conjugates showed a pattern very similar to fHbp natively expressed on bacterial surfaces or to the recombinant protein mixed with GMMA, respectively. However, the two conjugates elicited antibodies with similar serum bactericidal activity against meningococcal strains, superior to the protein alone or physically mixed with GMMA. Presentation of fHbp on GMMA strongly enhances the functional immune response elicited by the protein but its orientation on the bacterial surface does not have an impact. This study demonstrates the flexibility of the GMMA platform as a display and delivery system for enhancing antigen immunogenicity and further supports the use of such promising technology for the development of effective vaccines.

## 1. Introduction

Gram-negative bacteria spontaneously release outer membrane vesicles (OMVs) during growth. Generalized modules for membrane antigens (GMMA) are OMVs naturally shed from Gram-negative bacteria genetically modified to destabilize the linkage between the outer and the inner membrane and enhance OMVs production. GMMA represent a powerful technology for vaccine development, as they are simple to produce [1] but amenable to sophisticated manipulations. GMMA are produced with a high yield through a robust process, representing an attractive technology for low-cost vaccines [2]. Further mutations are introduced to reduce potential reactogenicity, in particular by modifying the structure of the lipid A component of lipopolysaccharide molecules, stimulator of the innate immune system [3]. GMMA can be also manipulated to up-regulate expression of homologous or heterologous antigens, increasing the breadth and magnitude of the immune response, with increased protection against different strains of the same bacterial species or against multiple species [4].

Multiple animal studies suggest that antigens displayed on OMVs induce more antigen-specific antibodies and/or antibodies with stronger functionality than corresponding recombinant protein formulations [3,4]. Reasons for this enhanced effectiveness could be related to different factors: (1) antigen particulation (GMMA have particle size in the range of 50–200 nm) facilitating uptake by antigen presenting cells (APCs) and/or follicular dendritic cells and increased germinal center reactions [5,6]; (2) high epitope density on GMMA; (3) presence of carrier T-helper epitopes; (4) intrinsic adjuvant properties (co-delivery of TLR2 and TLR4 agonists) [7,8]; (5) the fact that GMMA resembles the bacterial outer membrane, with protein antigens presented in their native environment and with their original conformation [9]. However, few experimental studies have been conducted to date to confirm these hypotheses and better elucidate the mode of action of OMV-based vaccines [10].

Here, we aimed to verify if antigen native orientation on GMMA plays a critical role in the antigen-specific humoral immune response elicited.

OMVs have been extensively studied as vaccine components against *Neisseria meningitidis* serogroup B (MenB) [11,12,13,14]. *Neisseria meningitidis* is a leading cause of bacterial meningitis worldwide and frequent epidemics in Sub-Saharan Africa [15,16]. Factor H binding protein (fHbp), an outer-membrane surface-exposed lipoprotein, expressed by almost all meningococcal strains, has been recognized as an important meningococcal virulence factor and included in licensed protein-based vaccines against group B meningococcus [17,18,19]. fHbp binds human factor H (fH), a negative regulator of the alternative pathway of the complement cascade, allowing meningococci to escape innate immunity [20,21]. Antibodies directed against fHbp are bactericidal and can both activate the complement cascade and block the recruitment of fH by bacteria [22,23,24,25,26].

It has been shown that fHbp on GMMA elicits antibodies in mice with broader bactericidal activity against African isolates than antibodies induced by recombinant fHbp [27,28].

We recently developed conjugation chemistries to easily and efficiently link polysaccharide antigens targeting both lipopolysaccharides and proteins on GMMA or linking protein antigens to the GMMA surface [29]. Indeed, we demonstrated that fHbp conjugated to the surface of GMMA from *Salmonella* Typhimurium enhanced the anti-fHbp IgG and functional response compared to the antigen alone or physically mixed with GMMA [29]. Here, we used similar conjugation tools [29,30] to conjugate fHbp antigen to resemble the native orientation of fHbp on the bacterial membrane or alternatively to randomly display fHbp on the membrane surface of GMMA. Such constructs were compared in mice with corresponding GMMA overexpressing fHbp. This study helped to elucidate the reasons related to the strong immune response induced by antigens when displayed on GMMA.

## 2. Results

### 2.1. Synthesis and Characterization of the Conjugates

#### 2.1.1. Characterization of fHbp Randomly Conjugated to GMMA

**Figure 1:**
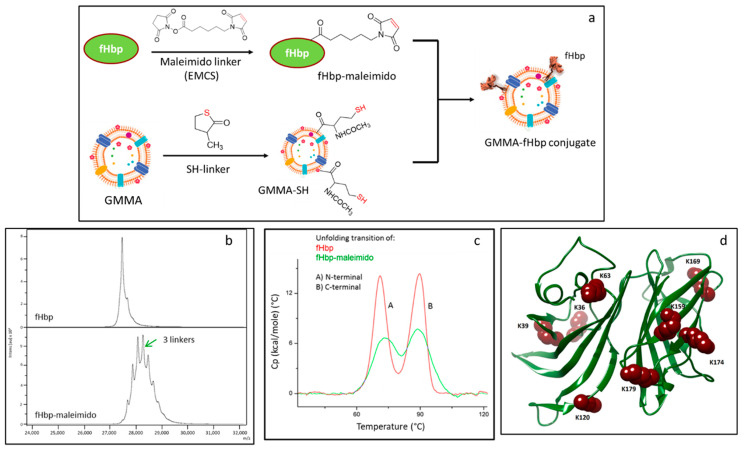
(**a**) Conjugation scheme for random linkage of fHbp to GMMA: fHbp was derivatized with EMCS linker followed by chemical conjugation to GMMA functionalized with SH linker; (**b**) MALDI-MS analysis of fHbp-maleimido compared to starting fHbp; (**c**) DSC analysis of fHbp-maleimido compared to starting fHbp; (**d**) fHbp structure highlighting lysine residues involved in the derivatization with EMCS linker as identified by peptide mapping analysis.

In a previous work, we generated GMMA-fHbp constructs consisting of *S*. Typhimurium GMMA with chemically conjugated fHbp using the chemistry shown in Figure 1a. Briefly, *Neisseria meningitidis* fHbp was randomly derivatized with N-ε-maleimidocaproyl-oxysuccinimide ester (maleimido linker, EMCS) before chemical conjugation to *S*. Typhimurium GMMA, previously activated with N-Acetyl-DL-homocysteine thiolactone (SH linker) [29] (Figure 1a). Derivatization conditions for fHbp were controlled in order to have a limited number of linkers per molecule, trying to minimize the impact on fHbp folding and conformation. Here, we first analyze further the *S*. Typhimurium GMMA-fHbp conjugate to understand further the effects of conjugation on protein conformation. MALDI TOF analysis confirmed an average of only three linkers per protein (Figure 1b); however, differential scanning calorimetry (DSC) indicated an impact on the fHbp conformation after linker introduction (Figure 1c). In fact, fHbp-maleimido showed the two expected N-terminal and C-terminal transitions at the same melting temperature (Tm) of underivatized fHbp [31], but the intensity of the peaks was strongly impacted. Analysis by peptide mapping [32] indeed confirmed random linkage of EMCS to fHbp, with eight lysine residues mainly involved in the derivatization, distributed in both domains of the protein: K36, K39, K63 and K120 of fHbp N domain and K159, K169, K174 and K179 belonging to the C-terminal fHbp domain [33] (Figure 1d).

#### 2.1.2. Synthesis of Selective and Random fHbp-GMMA Conjugates

We asked if by preserving fHbp conformation and native orientation on GMMA could have resulted in further improved immunogenicity and how much this feature can contribute to the strong immune response elicited by GMMA.

**Figure 2:**
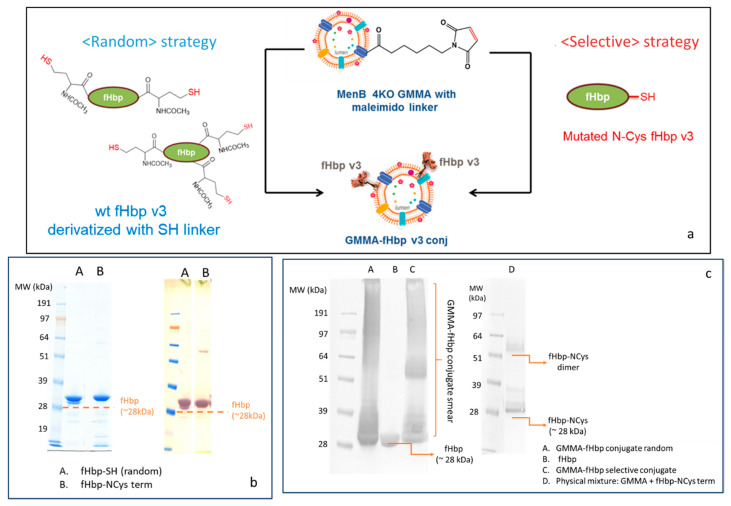
(**a**) Conjugation scheme for random linkage of fHbp to GMMA (fHbp derivatized with SH linker and chemically conjugated to GMMA functionalized with maleimido linker) or selective linkage of fHbp terminally mutated to have a Cys residue to GMMA-maleimido; (**b**) SDS-PAGE (left panel)/WB (right panel) analysis of fHbp randomly derivatized with -SH or terminally mutated with a Cys residue; (**c**) WB analysis of fHbp, random and selective conjugates and a physical mixture of GMMA with fHbp-NCys.

In order to preserve fHbp conformation and to verify if antigen orientation on GMMA could have an impact on the immune response elicited, fHbp was engineered to express a cysteine residue at the end of the N-terminus chain (fHbp-NCys). This protein was selectively linked to *Neisseria meningitidis* serogroup B (MenB) GMMA 4KO (also mutated to not express fHbp) that was previously activated with the maleimido linker (Figure 2a). This selective conjugation approach is potentially able to preserve the natural orientation of fHbp, mimicking the conformation of the protein on the MenB bacterial surface, as the linkage to the MenB GMMA surface only involves the first amino acid of the fHbp N-domain, resembling the way in which fHbp is naturally presented on bacterial membranes.

Similar chemistry was used to generate a random conjugate: this time, fHbp was randomly derivatized with the SH linker and attached to MenB GMMA 4KO derivatized with the maleimido EMCS linker (Figure 2a). MALDI MS analysis also confirmed the introduction of 2–3 linkers per fHbp molecul, while SDS-PAGE/Coomassie and Western blot (WB) analysis showed that no protein aggregation occurred after random SH linker introduction on lysine residues of fHbp (Figure 2b). Conjugate formation was verified by SDS-PAGE/WB. A diagnostic smear was detected in the lanes of both random and selective conjugates as a demonstration of the chemical linkage formation between fHbp and different protein components of the outer membrane surface of MenB GMMA, while fHbp alone or physically mixed with GMMA was detected as a single band (Figure 2c). Interestingly, when fHbp-NCys term was physically mixed with underivatized MenB GMMA 4KO, no smear was detected, indicating the lack of conjugation for the absence of a maleimido linker on the GMMA surface.

#### 2.1.3. Quantification of fHbp in Random or Selective GMMA Conjugates

The amount of fHbp linked to the GMMA surface in the two conjugates was estimated by competitive ELISA [34], working with anti-fHbp polyclonal mouse serum and using a GMMA naturally expressing fHbp and with a known amount of fHbp quantified by SRM mass spectroscopy for building the standard curve (Figure S2). The amount of conjugated fHbp was 3.4% and 3.6% with respect to the total protein in the random and selective conjugates, respectively. Such amounts were similar to that quantified in a MenB GMMA mutated to overexpress fHbp (GMMA OE fHbp). This GMMA had 2.0% fHbp with respect to the total protein amount by competitive ELISA, with a result that was in line with the quantification performed on the same construct by SRM mass.

### 2.2. Immunogenicity in Mice: Random and Selective Conjugates Compared to MenB GMMA Overexpressing fHbp

The selective conjugate, able to mimic the native orientation of fHbp on the bacterial surface, and the random conjugate, with fHbp having a random orientation on the GMMA surface, were tested in immunogenicity studies in mice in comparison to MenB GMMA overexpressing fHbp natively (GMMA OE fHbp), at a dose of 2.5 μg in terms of total protein. Two groups of mice received fHbp alone or physically mixed with GMMA at a dose of 1 μg.

**Figure 3:**
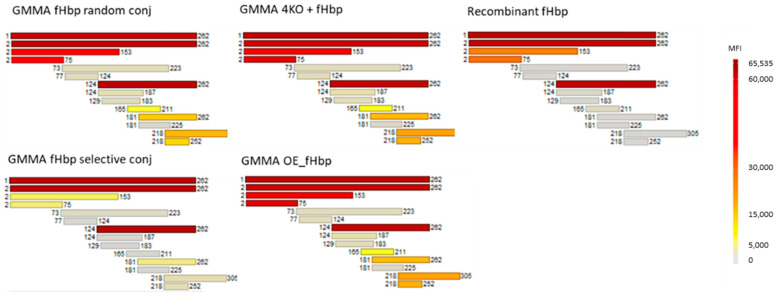
Protein microarray reactivity profile of pooled sera collected two weeks after the third immunization (day 35) with fHbp constructs, normalized according to ELISA titers. Each horizontal bar represents a single protein or protein fragment spotted in the microarray for fHbp v3.28 and color changes from light gray to dark red according to increasing mean fluorescence intensity (MFI) values, as shown in the vertical bar.

Sera collected two weeks after the third immunization at day 35 were pooled, normalized according to ELISA titers and tested in a protein microarray. In the protein array, full-length v3.28 and 12 fHbp v3.28 fragments were spotted on the chip. According to the results, sera from fHbp selectively conjugated to MenB GMMA showed a pattern very similar to antibodies elicited by fHbp natively expressed on GMMA (Figure 3). The antibodies were directed mainly towards the fHbp v3.28 C-terminal β-barrel domain.

Differently, sera from random conjugate immunization, fHbp alone or physically mixed with GMMA reacted strongly with the entire protein and smaller fragments of the N-term and C-term of fHbp v3.28 (Figure 3). It was noted that sera from formulations containing GMMA showed a broader epitope recognition with respect to fHbp alone.

These results confirmed that the selective approach, differently from the random one, was able to mimic the natural orientation of the protein on the GMMA surface, and that fHbp oriented on the GMMA via the N-terminus (natively or through selective conjugation) elicits greater antibody responses towards the out-facing C-terminal domain.

Looking at the post III (day 35) ELISA titers, both random and selective GMMA conjugates induced similar anti-fHbp IgG responses in mice (Figure S1).

A second dose-ranging study in mice was performed to better investigate the immune response elicited by the different constructs. All GMMA constructs, e.g., selective and random conjugates and GMMA OE fHbp, were tested at two different doses of 0.625 μg and 2.5 μg in terms of total protein. According to the results obtained in competitive ELISA, each mouse received a corresponding fHbp dose close to 0.02 μg and 0.1 μg, respectively. Two control groups of mice received fHbp alone or physically mixed with MenB GMMA 4KO at doses of 1 μg of fHbp and 2.5 μg of GMMA, corresponding to 10- or 40-fold higher doses with respect to fHbp in the GMMA constructs.

**Figure 4:**
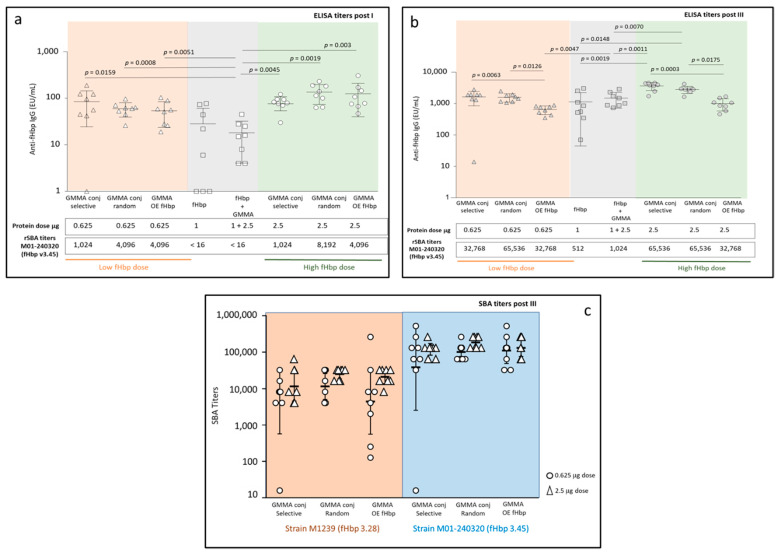
Immunogenicity study of random and selective GMMA-fHbp conjugates compared to GMMA OE fHbp, fHbp alone or physically mixed with GMMA 4KO. Eight 6-week-old female CD1 mice per group were i.p. immunized three times at days 0, 21 and 35. Summary graphs of anti-fHbp IgG response 3 weeks post first immunization (**a**) and 2 weeks after the third immunization (**b**): geometric mean units (bars) and individual antibody levels are reported together with corresponding serum bactericidal assay (SBA) titers of pooled sera for each group against M01-240320 MenB strain, expressing fHbp v3.45; (**c**) SBA titers of individual sera two weeks after the third immunization measured for selected groups against M01-240320 and M1239 MenB strains, expressing fHbp v3.45 and fHbp v3.28, respectively.

All constructs induced a significant anti-fHbp IgG response by 21 days after the first injection (Figure 4a). No statistical difference was observed among the three GMMA constructs, between the doses tested, at 21 days. At a low dose, all constructs elicited significantly higher anti-fHbp IgG responses than fHbp physically mixed with GMMA (*p* = 0.0159 for the selective conjugate, *p* = 0.0008 for the random conjugate, *p* = 0.0051 for GMMA OE fHbp). At a high dose, all GMMA constructs induced significantly higher antibody response than fHbp alone (*p* = 0.0045 for the selective conjugate, *p* = 0.0019 for the random conjugate and *p* = 0.003 for GMMA OE fHbp) or physically mixed with GMMA (*p* = 0.0003 for the selective conjugate, *p* = 0.0002 for the random conjugate and *p* = 0.0002 for GMMA OE fHbp), despite the lower content of fHbp in GMMA constructs with respect to the two control groups. Importantly, when tested for bactericidal activity against MenB strain M01-240320 expressing fHbp v3.45, sera elicited by the GMMA constructs were able to kill the bacteria after a single dose, differently from antibodies elicited by fHbp alone or mixed with GMMA (Figure 4a).

At day 49 (Figure 4b), two weeks after the third immunization, both at low and high doses, the two GMMA conjugates induced significantly higher responses than GMMA OE fHbp (at low dose, *p* = 0.0063 for the selective conjugate and *p* = 0.0126 for the random conjugate; at high dose, *p* = 0.0003 for the selective conjugate and *p* = 0.0175 for the random conjugate). At a low dose, no difference was identified in terms of anti-fHbp IgG response elicited by the GMMA constructs with respect to fHbp alone or physically mixed with GMMA, with the only exception represented by GMMA OE fHbp that elicited a significantly lower anti-fHbp IgG response than the physical mixture (*p* = 0.0047). At a high dose, both selective and random conjugates elicited significantly higher anti-fHbp IgG responses than fHbp alone (*p* = 0.0019 and *p* = 0.0148, respectively) or physically mixed with GMMA (*p* = 0.0011 and *p* = 0.0070, respectively), while the response elicited by the GMMA OE fHbp was not different with respect to the controls (fHbp alone or mixed with GMMA). Post III sera confirmed the ability of all three GMMA constructs to elicit higher SBA titers against MenB M01-240320 expressing fHbp v3.45 than fHbp alone or physically mixed with GMMA (from 32 to 128 times higher) (Figure 4b).

To better identify possible differences among the three groups immunized with GMMA, SBA analysis on post III sera was repeated on single sera (Figure 4c) against two different MenB strains, expressing fHbp v3.45 and fHbp v3.28. Statistical analysis confirmed no significant differences among the GMMA constructs.

## 3. Discussion

Over the last decade, outer membrane vesicles generated from Gram-negative bacteria manipulated to increase blebbing and reduce the potential reactogenicity associated with lipid A, here indicated as GMMA, have received greater attention as a platform for vaccine development [4,8,11]. GMMA are easy to manufacture and amenable to sophisticated manipulations. GMMA can be used against the bacterial pathogens from which they derive, manipulated to overexpress desired antigens to increase the breadth of protection and decorated with heterologous antigens resulting in multicomponent vaccines [10,29,35].

Through a large number of preclinical studies, it has been shown that GMMA are highly immunogenic. The reason for this could be linked to multiple factors not yet fully elucidated, including that GMMA present key antigens in their native conformation and orientation. As with many previous studies, in this study we have confirmed that *N. meningitidis* fHbp, when presented on the GMMA surface, is much more immunogenic than fHbp alone or simply physically mixed with GMMA [27,29,34,36]. Indeed, fHbp on GMMA elicited a higher antibody response than the controls despite a lower fHbp amount and, importantly, induced bactericidal titers post I, differently from the controls, and titers much higher than the controls after re-injection. We have already shown that fHbp localization on the GMMA surface is critical for induction of an effective immune response; in fact, when fHbp was expressed in the lumen of GMMA, the immune response elicited was weak and much lower compared to the recombinant fHbp [34]. Importantly, here we verified that the humoral response elicited by fHbp was similar independently of having the antigen natively expressed and presented on the bacterial surface (GMMA OE fHbp) or chemically conjugated to the GMMA surface by different chemistries. In particular, selective or random orientation of fHbp on GMMA did not change the functional immune response elicited and each conjugate induced similar levels of bactericidal activity towards strains expressing fHbp.

Protein array analysis allowed us to interrogate the location of the eliciting epitopes within the antibody responses to the fHbp antigen formulated as recombinant protein or displayed on GMMA through expression or conjugation technologies. Very interestingly, the results confirmed that different linkages of fHbp to GMMA resulted in exposure of different epitopes to the immune system, with the selective conjugate actually resembling native orientation of the lipoprotein on the bacterial surface, and resulting in elicitation of antibodies predominantly recognizing the C-terminus, while the random conjugate and free recombinant fHbp formulation elicited antibodies equally recognizing the N- and C- terminus. Therefore, results from this study indicate that, at least for fHbp, the native orientation of the protein antigen on GMMA can immune-focus the humoral response to the C-terminus, presumably the more exposed areas of the surface protein. The immunogenicity studies showed that immune-focusing by specific orientation or native expression is not a critical factor for the functional immune response elicited. This is in line with many studies identifying functional bactericidal epitopes of the protein in the N-terminus [37,38,39] in addition to the C-terminus and, importantly, the cooperativity of antibodies binding to the N-terminus or C-terminus has been well documented [40,41]. Therefore, while not significant for the fHbp protein, for other antigens, immune-focusing of responses to surface exposed functional epitopes by correct orientation on GMMA may have a more profound effect on immunogenicity.

One of the advantages indicated for the design of nanoparticle-based vaccines is that they can present multiple copies of subunit antigens in defined orientation that can potentially mimic their native conformation and natural host–pathogen surface interactions [42]. Combination of computational and structural biology allows the rational design of the well-oriented display of the most protective epitopes of a pathogen. Interestingly, orientation of antigen display on self-assembling protein nanoparticles has been shown to influence immunogenicity [43]. We cannot exclude that, based on the antigen investigated, retaining a native-like, unconstrained structure, allowing conformational epitopes to form, could have a positive impact on the immune response elicited and, actually, this could be critical for certain antigens. However, it seems clear from our study that other mechanisms would explain the “carrier effect” elicited by GMMA, already confirmed with a large number of different antigens (both proteins and polysaccharides) [29,30,44,45,46,47].

Here, we have shown how the chemical conjugation can represent a rapid and easy tool to explore the impact that certain variables can have on the immune response elicited by GMMA. Further than the impact of antigen orientation on the bacterial membrane, chemical conjugation was already used to investigate the impact of sugar length and density on the immune response elicited by GMMA constructs [48]. Additional studies are ongoing, trying to better elucidate the quality of antibodies generated by GMMA constructs and the mechanisms driving GMMA immune response [10], that could be instrumental to optimize the design of highly effective GMMA-based vaccines. This study demonstrates the flexibility of the GMMA platform and the use of adaptable conjugation or genetic engineering strategies for display and delivery systems for enhancing antigen immunogenicity.

## 4. Materials and Methods

### 4.1. GMMA Production and Purification

MenB GMMAs (produced from a 4 knock-out Δ*synX*, Δ*fHbp*, Δ*gna33*, Δ*lpxL*1 *Neisseria meningitidis* strain) were produced and characterized as previously described [29,49].

### 4.2. fHbp and fHbp-Cys Term Production, Purification and Characterization

fHbp v3.28 and the corresponding protein mutated to have a Cys residue at the N-terminus were expressed in *E. coli* as His-tag fusions [39]. Recombinant strains were first grown at 37 °C for 8 h in Luria broth medium containing 100 mg/L of ampicillin and then a dilution of 1:100 of the inoculum was transferred and grown in HTMC medium at 30 °C, 160 rpm for 26–30 h.

Cells were then collected by centrifugation at 3200× *g* for 20 min at 4 °C and resuspended in a 50 mM phosphate buffer, pH 8, containing 300 mM NaCl and a complete EDTA-free protease inhibitor (10 mL/g of biomass). Cells were disrupted by sonication (45′′ × 30 pulses, 40% amplitude, 15′′ pause between pulses). Debris and membrane were separated by centrifugation at 18,000 rpm for 1 h and then discarded.

The supernatants were purified by two chromatographic steps: Co^2+^ affinity (His GraviTrap TALON; GE Healthcare) and ENDOTRAP Red (Hyglos GmbH) for endotoxin removal. Recombinant proteins were purified in their soluble forms on a TALON column in a single-step elution using a 50 mM phosphate buffer, pH 8, containing 300 mM NaCl and 250 mM imidazole. Endotoxins were removed from the purified proteins with an ENDOTRAP Red pre-packed column after a buffer exchange (PBS 1X). The lipopolysaccharide content of the purified proteins was evaluated using the limulus amebocyte lysate test (Charles River).

Protein concentration was determined using the bicinchoninic acid (BCA) method (Pierce) and the Bradford method (Protein Assay; Biorad) using BSA as reference.

Purity of the purified proteins was evaluated by densitometry (SDS-PAGE Invitrogen, NuPAGE Novex 12% Bis-Tris Protein Gels in MES 1X, SimplyBlue SafeStain for gel staining and Phoretix 1D for gel analysis) and SE-UPLC (SE-UPLC BEH200 1.7 µm, flow 0.4 mL/mL, buffer 10 mM NaH_2_PO_4_, 400 mM (NH_4_)_2_SO_4_ pH 6).

### 4.3. Synthesis and Characterization of the GMMA Conjugates

#### 4.3.1. fHbp Derivatization with N-ε-malemidocaproyl-oxysuccinimde Ester (EMCS) Linker

EMCS linker as a 10 mg/mL solution in DMSO was added to 1.25 mg/mL of fHbp in PBS to have a 0.2:1 molar ratio of linker to Lys residues of the protein. The solution was mixed at room temperature for 4.5 h and then the derivatized protein was purified by chromatography on a PD10 column equilibrated with MES 10 mM pH 6. The resulting product was characterized by micro BCA (95% recovery), SDS-PAGE, DSC and MALDI-MS analyses [34]. Peptide mapping was used to identify amino acids involved in the derivatization [32].

#### 4.3.2. fHbp Derivatization with SH Linker (N-acetyl-DL-homocysteine Thiolactone)

fHbp at a concentration of 530 μg/mL in MES 30mM pH 6 was added of the activation buffer containing 2.6 mg/mL DTT, 13.16 mg/mL EDTA and 7.04 mg/mL N-acetyl-DL-homocysteine thiolactone in 100 mM borate pH 11 in order to have a 6.6-fold ratio of thiolactone to NH2 groups on fHbp. The solution was mixed at room temperature for 4 h and then the derivatized protein was purified by a PD10 desalting column (GE Healthcare Life Sciences) against 10 mM MES, 1mM EDTA pH 6. The resulting product was characterized by micro BCA (82% recovery).

#### 4.3.3. MenB GMMA Derivatization with N-ε-malemidocaproyl-oxysuccinimde Ester (EMCS) Linker

GMMA were resuspended at 4 mg/mL in 100 mM sodium phosphate (NaPi) pH 7.2 and EMCS linker (as a 50 mg/mL solution in dimethylsulfoxide (DMSO)) added to have a 0.6:1 molar ratio of linker to NH_2_ groups on GMMA. The reaction was mixed at room temperature for 4 h, GMMA-EMCS were purified by ultracentrifugation (110,000 rpm, 4 °C, 1 h) and derivatized GMMA were resuspended in 100 mM MES buffer pH 6. GMMA-EMCS were characterized by micro BCA (93% protein recovery) and TNBS and Ellman colorimetric methods for assessing whether 30% of NH_2_ groups were activated.

#### 4.3.4. Conjugations

Each protein, fHbp N-Cys and fHbp linker SH, was conjugated to GMMA-EMCS. Conjugation was performed in 100 mM MES pH 6 and both components were mixed at a final concentration of around 1 mg/mL. The reaction was mixed at room temperature for 4–5 h, the conjugate was purified by ultracentrifuge (110,000 rpm, 4 °C, 1 h) and resuspended in PBS.

Conjugates were characterized by micro BCA for total protein recovery and SDS-PAGE/Western blot analysis to confirm conjugate formation [29]. To quantify the amount of linked protein antigen, competitive ELISA was used, by building the calibration curve with a MenB GMMA overexpressing fHbp v3.28 [34].

### 4.4. Differential Scanning Calorimetry (DSC)

The thermal stability of fHbp proteins was assessed by differential scanning calorimetry (DSC) using a MicroCal VP-Capillary DSC instrument (Malvern). fHbp samples were prepared at a protein concentration of 0.5 mg/mL in 85 mM NaPi, pH 7.2. The DSC temperature scan ranged from 10 °C to 130 °C, with a 5 s filter period and a scan rate of 150 °C/h. Data were analyzed by subtraction of the reference data for a sample containing buffer only, and the curve-fitting procedure was performed using a 2-state model and the Levenberg/Marquardt (LM) non-linear least-square method, as provided within the Origin 7 software (OriginLab Corporation, Northampton, MA, USA).

#### 4.4.1. Immunogenicity Studies in Mice

All animal sera used in this study derived from mice immunization experiments performed at the GSK Animal Facility in Siena or at Toscana Life Sciences Animal Facility (Siena, Italy), in compliance with the relevant guidelines (Italian D. Lgs. n. 26/14 and European directive 2010/63/UE) and the institutional policies of GSK. The animal protocols were approved by the Animal Welfare Body of GSK Vaccines, Siena, Italy and by the Italian Ministry of Health (Approval number 804/2015-PR) and Animal Welfare Body of Toscana Life Sciences and by the Italian Ministry of Health (Approval number 479/2017-PR).

CD1 6-week-old female mice were immunized intraperitoneally (i.p.) three times at days 0 and 21 and 35. Anti-fHbp-specific IgG levels were measured post first and two weeks after the last injection by enzyme-linked immunosorbent assay (ELISA).

Microtiter plates were coated ON at 4 °C with 1 μg/mL purified fHbp. Wells were washed three times with PBS plus 0.1% Tween 20 (PBS-T) and blocked with 2.7% polyvinylpyrrolidone (PVP) for 2 h at 37 °C. After three washes with PBS-T, plates were incubated with mouse sera diluted 1:1000 for 2 h at 37 °C, followed by incubation with alkaline phosphatase-conjugated anti-mouse antibodies diluted 1:2000 in PBS-T plus 1% bovine serum albumin (BSA) for 90 min at 37 °C. Samples were incubated with p-nitrophenyl phosphate (SigmaFast OPD; Sigma, Darmstadt, Germany) at room temperature for 30 min, and the reaction was stopped with 4 N NaOH. Optical density was analyzed using a plate reader at a dual wavelength of 405/620 to 650 nm. The antibody titers of the single mouse serum samples are expressed in units ELISA per milliliter (UE/mL) and are calculated based on the standard curve.

SBA against serogroup B meningococcal M01-240320 and M1239 strains was performed using baby rabbit complement as complement source as previously described [50]. Briefly, bacteria were grown in Mueller–Hinton (MH) broth plus 0.25% glucose for approximately 1.5 h at 37 °C with shaking until early log phase (OD600) and then diluted in Dulbecco’s buffer (SIGMA) plus 1% BSA and 0.1% glucose (DPBS) to approximately 10^4^–10^5^ CFU/mL. Serum bactericidal titers were defined as the serum dilution resulting in a 50% decrease in the CFU/mL after 60 min of incubation of bacteria with the reaction mixture, compared to the control.

#### 4.4.2. Statistical Analysis

Datasets were analyzed using a non-parametric Mann–Whitney two-tailed test and Kruskall–Wallis test with Dunn’s post hoc analysis using Prism (GraphPad Software, San Diego, CA, USA). *p* values less than 0.05 were considered statistically significant.

### 4.5. Protein Array Analysis

Mouse sera were tested on protein microarrays containing recombinant full-length protein as well as overlapping fragments spanning the entire protein sequences of fHbp variant 3.28. The protein microarrays of 3 recombinant variant full-length antigens and fragments were generated as previously described [41]. Briefly, recombinant antigens were spotted on nitrocellulose-coated slides (FAST slides, Maine Manufacturing) using the no-contact Marathon Spotter (Arrayjet, Edinburgh, UK). Preliminary array validation was obtained by using the previously described approach. In particular, to confirm the efficiency and reproducibility of the protein deposition and immobilization on the chips, some test slides were probed with mouse anti-GST and anti-His6 polyclonal antibodies followed by detection with an AlexaFluor647-conjugated anti-mouse IgG secondary antibody (Jackson Immunoresearch, West Grove, PA, USA).

For mouse sample profiles, the experiments were performed as previously described [51]. Briefly, the assay was performed at room temperature and consisted in a two-step immunofluorescent assay. After a saturation step with Block-It (ArrayIt, Sunnyvale, CA, USA) for 1 h, plasma samples were diluted 1:500 in Block-It and incubated for 1 h prior to washing with PBS–Tween 0.1% (PBS-T) and incubating another hour with AlexaFluor647-conjugated rabbit anti-mouse IgG (Jackson ImmunoResearch). Then, after final washes with PBS-T, PBS and distilled water, fluorescence signals were detected by using the PowerScanner confocal laser scanner (Tecan Trading AG, Männedorf, Switzerland) and the 16-bit images were generated with the PowerScanner software v1.2 at a 10 μm/pixel resolution. Images were processed using the ImaGene 9.0 software (Biodiscovery Inc., EI Segundo, CA, USA). Microarray data analysis was performed using in-house developed software and R scripts. For each protein, the mean fluorescence intensity (MFI) of replicated spots was determined after subtraction of the background value surrounding each spot and the MFI of the corresponding tag. Signals were considered as positive when their MFI value was higher than 5000, corresponding to the MFI of protein spots after detection with rabbit AlexaFluor647-labeled anti-human antibody, plus 10 standard deviation values.

## Data Availability

The authors declare that data are contained within the article and in Supplementary Materials.

## Supplementary Materials

The following supporting information can be downloaded at: https://www.mdpi.com/article/10.3390/vaccines10081182/s1, Figure S1: Immunogenicity of random and selective GMMA-fHbp conjugates compared to GMMA OE fHbp, fHbp alone, GMMA 4KO alone or physically mixed with fHbp; Figure S2: Competitive ELISA to measure amount of fHbp in GMMA constructs.
