# Supplementary material for: Investigating the Role of Antigen Orientation on the Immune Response Elicited by Neisseria meningitidis Factor H Binding Protein on GMMA
Source: Vaccines (Basel). 2022 Jul 26;10(8):1182. doi: 10.3390/vaccines10081182 (PMC9331691; doi:10.3390/vaccines10081182)
Supplement: Supplementary file 1 [file vaccines-10-01182-s001.zip › vaccines-1804430-supplementary.pdf]

vaccines

MDPI

Supplementary Information

Investigating the Role of Antigen Orientation on the Immune Response Elicited by *Neisseria meningitidis* Factor H Binding Protein on GMMA

Renzo Alfini [1], Brunella Brunelli [2], Erika Bartolini [2], Martina Carducci [1], Enrico Luzzi [2], Francesca Ferlicca [2], Scilla Buccato [2], Barbara Galli [2], Paola Lo Surdo [2], Maria Scarselli [2], Giacomo Romagnoli [2], Elena Cartocci [2], Domenico Maione [2], Silvana Savino [2], Francesca Necchi [1], Isabel Delany [2] and Francesca Micoli [1,*]

[1] GSK Vaccines Institute for Global Health (GVGH), 53100 Siena, Italy; renzo.x.alfini@gsk.com (R.A.); martina.x.carducci@gsk.com (M.C.); francesca.x.necchi@gsk.com (F.N.)

[2] GSK, 53100 Siena, Italy; brunella.x.brunelli@gsk.com (B.B.); erika.x.bartolini@gsk.com (E.B.); enrico.x.luzzi@gsk.com (E.L.); francesca.x.ferlicca@gsk.com (F.F.); scilla.x.buccato@gsk.com (S.B.); barbara.x.galli@gsk.com (B.G.); losurdopaola7@gmail.com (P.L.S.); maria.x.scarselli@gsk.com (M.S.); giacomo.x.romagnoli@gsk.com (G.R.); elena.x.cartocci@gsk.com (E.C.); domenico.x.maione@gsk.com (D.M.); silvana.x.savino@gsk.com (S.S.); isabel.x.delany@gsk.com (I.D.)

* Correspondence: francesca.x.micoli@gsk.com

Citation: Alfini, R.; Brunelli, B.; Bartolini, E.; Carducci, M.; Luzzi, E.; Ferlicca, F.; Buccato, S.; Galli, B.; Lo Surdo, P.; Scarselli, M.; et al. Investigating the Role of Antigen Orientation on the Immune Response Elicited by *Neisseria meningitidis* Factor H Binding Protein on GMMA. *Vaccines* **2022**, *10*, 1182. https://doi.org/10.3390/vaccines10081182

Academic Editor: Giampiero Girolomoni

Received: 21 June 2022
Accepted: 16 July 2022
Published: 26 July 2022

Publisher's Note: MDPI stays neutral with regard to jurisdictional claims in published maps and institutional affiliations.

Copyright: © 2022 by the authors. Submitted for possible open access publication under the terms and conditions of the Creative Commons Attribution (CC BY) license (https://creativecommons.org/licenses/by/4.0/).

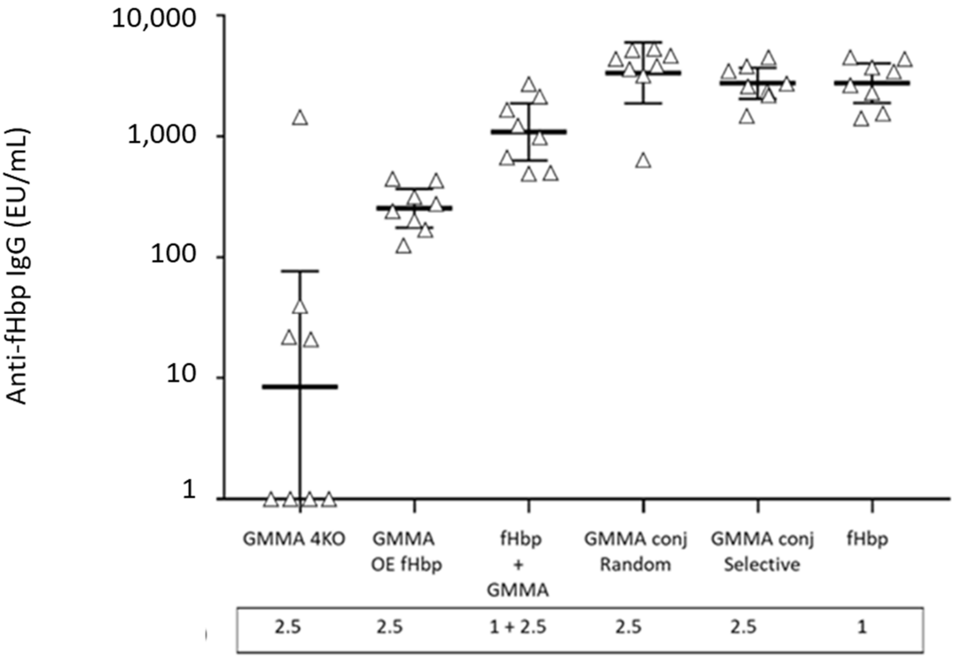

Figure S1. Immunogenicity of random and selective GMMA-fHbp conjugates compared to GMMA OE fHbp, fHbp alone, GMMA 4KO alone or physically mixed with fhbp. Eight CD1 mice 6-week-old female per group were i.p. immunized three times at days 0, 21 and 35. Summary graph of anti-fHbp IgG response, reporting geometric mean units (bars) and individual antibody levels (triangles) 2 weeks post third immunization.

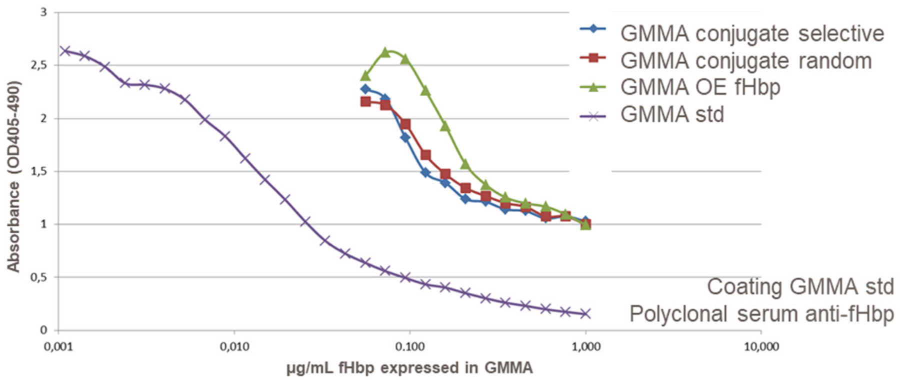

Figure S2. Competitive ELISA to measure amount of fHbp in GMMA constructs. A standard curve was built by spiking an anti-fHbp polyclonal mouse serum with known quantity of GMMA overexpressing fHbp (GMMA std) quantified by SRM mass spectroscopy, starting from a known concentration of 1 µg/mL fHbp and serially diluted up to 0.001 µg/mL fHbp. The two fHbp-GMMA conjugates and GMMA OE fHbp were tested in the same conditions and quantified in terms of fHbp amount respect to the total protein by interpolation to the standard curve.
